# A narrative review on fecal microbiota transplantation routes in ulcerative colitis: identifying the optimal approach across key parameters

**DOI:** 10.1097/MS9.0000000000003841

**Published:** 2025-09-02

**Authors:** Rohan Singhal, Gayatri Ghadvaje, Nanditha Karra, Sai Teja Gadde, Prerna Chandra, Bharat Krishna Teja Voruganti, Navya Pillikunte Doddareddy, Sadaf Iftikhar, Tirath Patel

**Affiliations:** a Department of Medicine, Atal Bihari Vajpayee Institute of Medical Sciences and Dr. Ram Manohar Lohia Hospital, New Delhi, India; b Faculty of General Medicine, Smolensk State Medical University, Smolensk, Russia; c Department of Medicine, Osmania Medical College, Hyderabad, India; d Department of Medicine, All India Institute of Medical Sciences (AIIMS), Mangalagiri, India; e Department of Medicine, Deccan College of Medical Sciences, Hyderabad, India; f Department of Medicine, Gandhi Medical College, Hyderabad, India; g Department of Medicine, Bangalore Medical College and Research Institute, Bangalore, India; h Department of Medicine, Akhtar Saeed Medical and Dental College, Lahore, Pakistan; i Department of Medicine, Trinity Medical Sciences University School of Medicine, Saint Vincent and the Grenadines

**Keywords:** chronic inflammatory bowel disease, fecal microbiota transplantation, gut microbiota, long-term outcomes, ulcerative colitis

## Abstract

Fecal microbiota transplantation (FMT) has gained increasing attention as a novel therapeutic approach for treating ulcerative colitis (UC), a chronic inflammatory bowel disease that causes an imbalance in the gut microbiota. Although FMT has demonstrated the potential to induce remission in UC patients, the most effective route of administration remains an area of active investigation. This narrative review provides a comprehensive comparison of different FMT delivery methods, such as oral capsules, enemas, colonoscopy, and nasogastric or nasoenteric tubes, across a range of clinically relevant parameters, including efficacy, safety, patient satisfaction, microbiota changes, pretreatment protocols, and cost-effectiveness. Furthermore, we examined how post-FMT dietary interventions may influence microbial engraftment and improve the long-term outcomes in patients with UC. In addition to assessing these practical and clinical factors, this review highlights the importance of patient-centered considerations, such as the tolerability and convenience of each administration route. The integration of these findings can provide valuable insights into how different FMT routes affect disease outcomes and guide clinicians in optimizing the treatment for individual patients. By synthesizing current evidence on these key variables, we aimed to identify the most effective and feasible FMT approach for UC. Establishing standardized protocols for FMT administration, informed by this analysis, will be crucial for ensuring consistency in clinical practice, improving patient outcomes, and minimizing adverse events. The insights from this review will help pave the way for more targeted and individualized FMT strategies, ultimately enhancing the therapeutic landscape of UC management.

## Introduction

Ulcerative Colitis (UC), the most prevalent form of Inflammatory Bowel Disease (IBD), is an idiopathic condition characterized by erosion of the colonic wall, leading to mucosal and submucosal inflammation, and frequent bleeding. This chronic disorder often initiates in the rectum and progresses proximally, impacting both physical and mental health^[1]^.

The highest incidence rates of UC have been reported in Northern Europe and North America, with a bimodal age distribution, peaking between ages 15 and 30 and 50–70 years. Clinically, UC typically presents with bloody diarrhea, abdominal pain, fever, weight loss, tenesmus, and general malaise. The diagnosis is best confirmed by colonoscopy with biopsy in conjunction with clinical findings. Microbiological tests are essential to exclude bacterial or parasitic infections.

During acute exacerbations, patients may exhibit an elevated erythrocyte sedimentation rate (ESR), C-reactive protein (CRP) level, and leukocytosis. A “stove pipe” sign on barium enema may be seen in long-standing cases, although it is not diagnostic. Elevated levels of P-ANCA and ASCA are sometimes present but are not specific to UC^[2]^. Multiple biopsies and endoscopic evaluation are vital for accurate diagnosis and management. Treatment plans should consider disease extent, severity of inflammation, relapse frequency, prior treatments, symptom severity (e.g., stool frequency and rectal bleeding), and patient preferences^[3]^. This approach supports the staging of proctitis, extensive colitis, and fulminant colitis, which in turn guides treatment^[4]^. Due to the multifactorial nature of UC, effective treatment requires a comprehensive approach that integrates dietary modifications, probiotics, prebiotics, antibiotics, and fecal microbiota transplantation (FMT)^[5]^.

For proctitis, topical 5-Aminosalicylic acids (5-ASA) and/or corticosteroids are effective, while systemic and topical 5-ASA combined with oral corticosteroids is preferred for extensive disease. In severe cases, intravenous corticosteroids and immunosuppressants, such as Azathioprine and Methotrexate, are required for remission induction and maintenance^[6]^. Colectomy is indicated in fulminant colitis, toxic megacolon, or when conservative treatment fails^[7]^. FMT, involving the introduction of fecal material from a healthy donor into a recipient’s gastrointestinal tract, has been effective in treating recurrent *Clostridium difficile* infections and shows promise for UC^[8]^. By promoting microbial diversity and restoring immune homeostasis, FMT may reduce colonic inflammation, as studies suggest it reestablishes CD4+, Treg cells, enhances IL-10 production, and restores intestinal memory/effector T-cell populations^[5]^. Indications for FMT include moderate to severe cases with frequent relapses or those unresponsive to immunosuppressive therapy^[9]^.

FMT can be administered via the upper or lower gastrointestinal route. Upper GI delivery includes donor stool infusion via gastroscope, nasogastric, or nasojejunal tubes, or oral capsules, while lower GI delivery uses colonoscopy, sigmoidoscopy, or retention enemas^[10]^.

**Figure 1.**
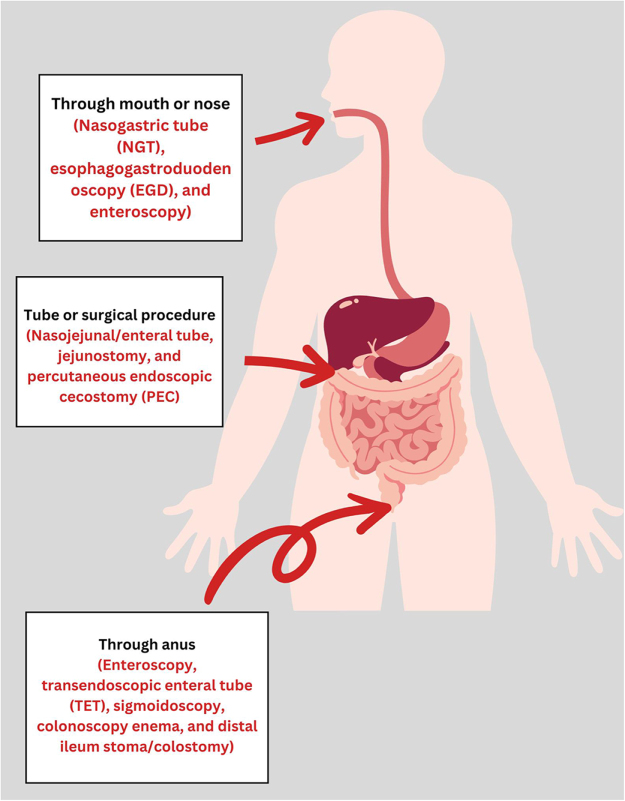
FMT administration routes.

Figure 1 provides a clear and concise overview of the various FMT administration routes used in the treatment of UC. It features three primary methods for delivering fecal microbiota: oral capsules, where patients swallow capsules containing donor stool; intestinal delivery via nasogastric or nasoenteric tubes, which deliver fecal material directly into the small intestine; and anal delivery, where fecal microbiota is introduced into the colon through an enema or colonoscopy.

This study aimed to compare these FMT administration methods, considering multiple parameters including efficacy, safety, pretreatment protocols, microbiota changes post-FMT, patient satisfaction, cost-effectiveness, and dietary interventions following FMT, contributing to an emerging area of research on comprehensive FMT management in UC.

## Methodology

This narrative review synthesized data from various primary studies to evaluate the efficacy, safety, and practical considerations of different fecal microbiota transplantation (FMT) administration routes for the treatment of ulcerative colitis (UC). The review was based on a comprehensive search of the PubMed, PubMed Central, and Google Scholar databases, which were conducted by all authors.

### Search strategy and inclusion criteria

We used a broad search strategy to identify studies that assessed the effectiveness of FMT routes in patients with UC. The search included keywords such as “fecal microbiota transplantation,” “ulcerative colitis,” “efficacy,” and “safety.” We included only human studies published in English, and excluded animal studies, non-English articles, and duplicate publications. The selection focused on studies that directly compared different FMT delivery methods, including oral capsules, enemas, colonoscopy, and nasogastric/nasoenteric tubes.

**HIGHLIGHTS**

- Comparison of FMT routes: The review systematically evaluates different FMT delivery routes, including colonoscopy, enema, and oral capsules, focusing on efficacy, safety, patient satisfaction, pretreatment protocols and patient satisfaction, aiding clinicians in selecting the most appropriate method for each patient.
- Development of standardized protocols: The findings emphasize the need for standardized protocols in FMT administration for UC, addressing pretreatment, route selection, and dietary interventions to enhance consistency and effectiveness across clinical settings.
- Cost-effectiveness of FMT options: The review highlights that oral capsules provide a cost-effective FMT option, potentially lowering the financial burden on patients and providers without compromising treatment efficacy.
- Role of post-FMT dietary interventions: Post-FMT dietary strategies, such as low-FODMAP diets and the inclusion of prebiotics and probiotics, are shown to support microbial engraftment and improve long-term remission rates.
- Directions for future research: The review identifies key areas for further investigation, such as optimal FMT frequency, microbiota matching, donor selection, and the integration of FMT into routine care, which could refine treatment protocols and enhance patient outcomes in UC management.

### Study selection and data extraction

The authors reviewed relevant articles, focusing on clinical trials, systematic reviews, and meta-analyses that reported on at least one of the following parameters: clinical efficacy, safety, patient satisfaction, microbiota changes, pretreatment protocols, and cost-effectiveness. All the included studies were rigorously evaluated for methodological quality.

Disagreements in article selection or data interpretation were resolved through consensus discussions among the authors. The final dataset was compiled into tables presenting the study characteristics, delivery routes, efficacy outcomes, and reported adverse events.

### Data presentation and analysis

Tables 1 and 2 present the articles identified from the databases. Table 1 includes systematic reviews and meta-analyses that compared different FMT routes across various patient populations. Studies were categorized by the delivery method (upper gastrointestinal route vs. lower gastrointestinal route) and their respective follow-up periods. We also reviewed the patient satisfaction, tolerability, and practical considerations for each FMT route.

**Table 1:** Systematic reviews and meta-analysis

Authors	Sample size	Delivery route	Time of follow-up	Pretreatment/concomitant medications (if any)	Efficacy	Adverse events
Liu *et al*	292	Upper GIT (naso-duodenal infusion or oral capsules) vs. lower GIT (colonoscopy infusion and enema)	Week 7–12	Bowel lavage, pre-antibiotic pretreatment	FMT delivered via lower GI route was superior to upper GI route about combining clinical remission with endoscopic remission/response	6.8% of patients assigned to FMT group reported SAEs compared with 4.8% allocated to the control group
Wei *et al*	425	Upper GIT (naso-duodenal infusion or oral capsules) vs. lower GIT (colonoscopy infusion and retention enema)	Week 7–12	Pre-antibiotics given in only 2/9 RCTs	Clinical remission was better when the FMT delivery route was via the lower GIT	The FMT group had a higher number of SAEs than the control group; however, in the sensitivity analysis, the difference in the SAEs was not significant
Zhao *et al*	959	Colonoscopy, retention enema, nasojejunal or nasogastric tube, duodenal infusion via upper endoscopy, and orally administered capsules	Week 4–18 months	Metronidazole/vancomycin/rifaximin	Administration of FMT via the lower GI tract was more effective in achieving CR than via the upper GI tract (44.3% vs. 31.7%)	The overall incidence of serious adverse events of FMT was 5.9%
Feng *et al*	580	Upper GIT (naso-duodenal infusion or oral capsules) vs. lower GIT (colonoscopy infusion and enema)	Week 7–48	-	The non-oral capsule group had a 25.61% remission rate for UC, while the oral capsule group showed no significant difference from the control (46.67%). Oral capsules provided better clinical	No significant difference in the incidence of adverse reactions between the FMT group and control group.
Costello *et al*	445	Upper GIT (nasogastric/nasojejunal tube or endoscopic duodenal infusion) vs. lower GIT (enema, colonoscopy or rectal tube)	Month 1–Month 72	Metronidazole/vancomycin/rifaximin/amo xicillin/thiopurines/mesalamine/corticosteroids/glucocorticoid, 5-ASA/anti-TNF/prednisol one	CR rates were (63%) for upper GI delivery vs. (53%) for lower GI delivery. Clinical remission rates were (21%) for upper GI delivery and (19%) for lower GI delivery	No significant differences between number or type of adverse event between donor and placebo groups in either study
Tang *et al*	431	Upper GIT (FMT capsules, nasoduodenal tube) vs. lower GIT (colonoscopy infusion and retention enema)	Week 7–48	Mesalamine/PEG bowel preparation/int estinal lavage/corticosteroids/Glucocorticoid/5-ASA/anti-TNF/thiopurin es/methotrexa te/prednisone/azathioprine	The CR rate was 50.8% for FMT via lower GIT and 31.4% for placebo, showing better efficacy for FMT. For FMT via upper GI, the rates were 30.0% and 30.3%, with no significant difference between groups.	The incidence of adverse reactions was 12.4% in the FMT group and 11.2% in the placebo group; the difference between groups was not significant
El Hage *et al*	324	Upper GI tract (through nasoduodenal infusions or capsules) or lower GI tract (through colonoscopic infusions or enemas)	Week 7–12	Mesalamine/a nti-TNF/immunos uppressants/g lucocorticoids/preantibiotics	The differences in clinical remission rates in delivery through the upper or lower GI tract or upper and lower GIT were not statistically significant.	SAEs occurred in 8.07% of the FMT group and 6.13% of the placebo group. Rates of worsening colitis, colectomy, and CDI were similar between both groups.
Narula *et al*	277	Upper GIT (naso-duodenal infusion) vs. Lower GIT (colonoscopy infusion and enema)	Week 7–12	5-ASA/thiopurine/anti-TNF/vedolizumab/prednisolone/ methotrexate/steroids	The clinical remission rate for FMT via lower GIT was 44.4% compared to 20.5% for placebo, showing better efficacy for FMT. For upper GI, the rates were 30.4% and 32%, with no significant difference.	No statistically significant increase in serious adverse events with FMT compared to controls
Cao *et al*	446	Upper GIT (naso-jejunal tube, nasogastric tube) vs. Lower GIT (colonoscopy and enema)	Month 1–Month 12		Rate was 33.37% for the enema group, 25.74% for the colonoscopy group, and 15.98% for other routes (e.g., nasogastric, nasojejunal, gastroscopy). The efficacy of colonoscopy was like enema.	NR
Ding *et al*	109	Midgut TET vs. colonic TET	1–5 years	5-ASA	No difference in efficacy was noted between patients who were administered from the mid-gut and from colonic TET	FMT-related adverse events were observed in 17.4% of procedures including one SAE (myasthenia gravis) and 56 non-serious adverse events
Sun *et al*	133	Upper gastrointestinal delivery (nasogastric/nasojejunal tube and gastroscopy) versus lower gastrointestinal delivery (colonoscopy and/or enema)	Month 1–Month 18	Bowel lavage, antibiotics, probiotics/rifaximin/vancomycin/metoclopramide/omeprazoleasa/azathioprine/methotrexate/mesalazine/ga tifloxacin/infliximab/gentamicin/norfloxacin/prednisolone	The rate of CR in patients with the upper GI delivery was 27.5% whereas in lower GI delivery, it was 29.8%; no difference in CR was detected	All studies reported mild adverse events(including self-limiting fever, abdominal discomfort, abdominal pain, bloating, diarrhea, and vomiting)
Imdad *et al*	277	Nasoduodenal vs. rectal route	Week 7–12 Month	Mesalamine	The authors were not able to make any conclusive recommendation	Unclear whether there is a difference in SAE rates between the intervention and control groups
Shi *et al*	234	Upper (NG tube, gastroscopy) vs. lower GI (colonoscopy,sigmoidoscop y, enema) infusions	Week 4–18 months	Lavage with PEGsolution/omep razole/metroni dazole/metoclopramide/rifaximin|mesala mine/anti-TNF/glucocorti coids/methotr exate	UGI infusions showed a remission rate of 17% while lower GI infusions showed a remission rate of 36%	AEs were monitored and reported in 19 of 25 studies. The majority of adverse events were slight and self-resolving including bloating, abdominal pain, cramping, blood in stool, diarrhea and fatigue.

**Table 2:** Study characteristics of RCTs (randomized controlled trials)

Authors	Disease severity	Sample size	Delivery route	Follow-up period	Pretreatment	Efficacy CR	Adverse events
Sarbagili, 2022	Active UC(SCCAI5–11 and endoscopic Mayo score of 2–3)	19/15	Colonoscopy and retention enema	2 months	Bowel lavage 2–3 L macrogol solution	4|6	Abdominal pain, diarrhea, chills
Costello, 2019	Mid to moderate (Mayo score of 3–10 and endoscopic subscore ≥2)	38/35	Colonoscopy and retention enema	12 months	3 L of polyethylene e glycol bowel preparation	18|6	Worsening of colitis, pneumonia
Haifer, 2022	Mid to moderate (Mayo score of 4–10 and endoscopic Mayo subscore ≥1)	15/20	Oral lyophilized FMT	11 months	Amoxicillin, metronidazole, and doxycycline	11|5	Mild self-limiting gastrointestinal complaints(placebo), worsening of colitis (FMT group)
Moayyedi, 2015	Mid to moderate (Mayo score of 4–10 and endoscopic Mayo subscore ≥1)	38/37	Retention enema	1.5 months	NA	9|2	Worsening of colitis, rectal abscess
Rossen, 2015	Mild to moderate (SCCAI 4–11,and endoscopic subscore ≥1)	23/25	Nasoduodenal infusions	3 months	Bowel lavage 2 L macrogol solution	7|8	Vomiting, fever, increased stool frequency.
Crothers, 2021	Mild to moderate (Mayo score of 4–10; endoscopic Mayo subscore ≥1, a rectal bleeding subscore ≥1, and a stool frequency subscore ≥1)	6|6	Colonoscopy followed by oral frozen encapsulated cFMT	9 months	Standard bowel prep before colonoscop y	2|0	Fever and worsening of disease
Botacui, 2015	Moderate to severe (Montreal classification of severity of UC: S2 and S3)	15	Endoscopic infusion tube through gastroscope channel into distal duodenum	3 months	Metoclopra mide 10 mg IM and esomopraz ole 40 mg IV 1 hour prior to FMT	8|14	Fever, diarrhea; mild testicular pain in one patient. No severe adverse events
Paramsothy, 2017	Mild to moderate (Mayo score of 4–10)	41/40	Colonoscopy and retention enema	2 months	No pre-FMT therapy	18|8	NR
Kunde, 2013	Mild to moderate (PUCAI 15-65)	10	Enema	1 month	NR	3	Bloating, diarrhea, cramping, fullness, flatulence, hematochezia, fever, chills
Angelburger, 2013	Moderate to severe (Mayo score ≥6)	5	Nosojejunal + enema	3 months	Bowel lavage, antibiotics	0	Vomiting, fever e, sore throat, flatulence
Damman, 2015	Mild to moderate (UCDAI between 3 and 10)	7	Colonoscopy	3 months	Bowel lavage	0	Abdominal cramping, pain and stool output immediately after FMT
Kump, 2013	Moderate to severe (Mayo score between 8 an 110)	6	Colonoscopy	12 months	PEG colonic lavage	0	Fever
Chen, 2020	Moderate to severely active UC (Mayo score between 6 and 12)	9	Nasojejunal tube (NJT) or transendoscp ic enteral tubing (TET)	Week 2, 12	Mesalamine (oral)/Steroids (intravenous s)	5|9	Mild adverse events seen in 3 patients including mild abdominal pain, diarrhea and fatigue in patients who received therapy via NJT

## Discussion

### Efficacy

FMT shows variable effectiveness in UC treatment, influenced by factors such as delivery method, follow-up duration, and disease severity. Studies have indicated higher remission rates with methods such as colonoscopy and retention enemas. Sarbagili *et al* reported moderate success with these methods, while Costello *et al* observed better long-term outcomes, likely due to a thorough bowel preparation^[11,12]^. Non-invasive methods, such as oral lyophilized FMT, also showed promise^[13]^, although nasoduodenal routes generally resulted in lower remission rates^[14]^.

Moayyedi *et al* found that retention enema FMT induced significant remission, with better outcomes linked to single-donor stool and a shorter disease duration^[15]^. Oral frozen encapsulated FMT (cFMT) also showed potential, although issues around home storage and colon effects need further study^[16]^.

Damman *et al* found that a single colonoscopic FMT provided only brief remission for one patient with UC, with no lasting improvements in others^[17]^. Similar studies also reported only short-term benefits but no sustained remission from a single treatment^[18]^.

These findings underscore that effective FMT treatment for UC requires careful consideration of the delivery method, disease severity, and prolonged follow-up. Direct delivery methods like colonoscopy and enema, supported by repeated or long-term treatment plans, appear more promising for better remission outcomes. Optimizing these variables could make FMT a more reliable treatment option for UC.

FMT via percutaneous endoscopic cecostomy (PEC) is emerging as a promising modality for UC, especially in steroid-dependent patients unresponsive to standard therapies. PEC delivers fecal microbiota directly into the cecum through a tube in the abdominal wall, allowing donor microbes to be evenly distributed throughout the colon and closely mimicking natural gut flow. This approach restores microbial diversity and balance in the colon, reducing inflammation and supporting intestinal health. Lower GI routes like PEC are more effective because they target the most affected areas and ensure sustained colonization of beneficial bacteria^[19]^.

Daily or twice-weekly FMT via PEC is effective because frequent administration promotes persistent engraftment of donor microbes, maximizes anti-inflammatory effects, and helps maintain remission, benefits supported by studies showing improved outcomes with multi-session and higher-intensity FMT regimens. In a recent case, a 24-year-old man with recurrent UC received daily FMT through PEC for 1 month in the hospital, followed by twice-weekly treatments at home for three months, resulting in significant symptom improvement and over a year of remission^[19]^. Additionally, a study^[20]^ found that FMT via transendoscopic enteral tubing (TET) (another lower GI route) had better response rates at 2 and 12 weeks compared to nasojejunal tubing (NJT), further underscoring the advantage of direct colonic delivery for optimal clinical outcomes.

Liu *et al* showed that lower GI routes, like colonoscopy and enemas, achieved better remission than upper routes, especially with pooled donor stool and higher FMT frequency^[21]^, a finding echoed by Wei *et al*^[22]^. Other studies also confirmed that lower GI routes generally provide more effective results, particularly with higher doses of FMT, with no significant impact from using multiple versus single donors, fresh versus frozen stool, or antibiotic pretreatment^[23,24]^.

The optimal method for FMT in UC remains uncertain, with some studies reporting comparable remission rates between the upper and lower GI routes^[25–27]^, while others could not draw definitive conclusions^[28]^.

Ding *et al* found no difference in efficacy between FMT administered via the midgut and colonic TET^[29]^. Tang *et al* reported that using frozen stool from multiple donors via the lower GI tract was more effective than single-donor stool via the upper GI tract, a finding also supported by Narula^[30,31]^. Cao *et al* reported similar remission rates for enema (33.37%) and colonoscopy (25.74%), both of which were more effective than other methods^[32]^.

Gastric acid may impair the growth of certain gut bacteria, leading to lower response rates with upper GI delivery due to the acidic environment^[33]^. The use of proton pump inhibitors (PPIs) may help neutralize gastric acid, creating a more favorable environment for transplanted bacteria to survive and colonize the GI tract^[34]^. However, current evidence does not support a clinically significant benefit from routine PPI use in upper GI FMT protocols. Studies have found no significant difference in FMT efficacy between patients who received PPIs and those who did not, suggesting that the theoretical benefit of improved bacterial survival may be offset by negative effects associated with PPIs^[35]^. As such, the use of PPIs in this context remains largely theoretical, and further research is needed to determine their role in improving clinical outcomes in upper GI FMT recipients

### Safety

FMT has gained attention as a potential therapy for UC, though its safety profile varies depending on administration methods, patient characteristics, and disease severity.

Studies on mild to moderate UC cases, like those by Sarbagili *et al*, Rossen *et al*, and Kunde *et al*, report common, mild AEs (e.g., abdominal pain, diarrhea, and fever) from colonoscopy, nasoduodenal infusions, and enemas^[11,14,36]^. These symptoms were mostly transient and self-limiting without significant complications, indicating good tolerability in mild to moderate cases.

Conversely, more serious adverse events were noted by Costello *et al* and Moayyedi *et al*^[12,15]^. Both studies, which examined FMT via colonoscopy and retention enema, documented cases of worsening colitis, Costello *et al* additionally reported pneumonia, and Moayyedi *et al* described a rectal abscess. These results suggest that more aggressive or prolonged treatments may elevate the risk in patients with UC, despite some therapeutic benefits.

Oral FMT has also been investigated. Haifer *et al* found that while the placebo group experienced mild symptoms, patients receiving oral lyophilized FMT reported worsening of colitis, suggesting that even noninvasive methods can pose significant risks^[13]^. Similarly, Crothers *et al*, who combined colonoscopy with oral frozen encapsulated FMT, reported fever and worsening disease, raising caution for combined approaches^[16]^.

In studies of more severe UC cases, Cui *et al* and Paramsothy *et al* used endoscopic infusion and colonoscopy, respectively^[37,38]^. Both patients reported mild AEs, such as fever and diarrhea, with no major complications. This suggests that, with adequate preparation, FMT may be safer in patients with severe UC. Angelburger *et al* noted similar findings, but reported vomiting and fever with a combination of nasojejunal and enema delivery, emphasizing the importance of carefully managing patients undergoing combination therapies^[39]^.

Studies by Damman *et al* and Kump *et al*, using colonoscopy for FMT, observed mild AEs such as abdominal cramping and fever, with no severe AEs, supporting the safety of colonoscopy-administered FMT in most cases^[17,18]^. Finally, Chen *et al*, who studied FMT delivered via nasojejunal or transendoscopic enteral tubing, found only mild AEs, such as diarrhea and abdominal pain, suggesting that even in severe UC, FMT may be relatively safe with appropriate protocols^[20]^.

Therefore, although mild gastrointestinal AEs like diarrhea and bloating are commonly reported, more severe complications such as worsening colitis, pneumonia, and abscesses have also occurred, particularly in patients receiving aggressive treatments^[12,15]^. These studies underscore the need for individualized FMT approaches, careful monitoring, and patient selection to minimize risks and optimize safety outcomes.

Initial results from FMT via the PEC method are promising, but precautions must be taken to prevent complications such as hematoma, infection, and pressure necrosis, requires proper device placement and regular postoperative monitoring, which necessitates the need for more randomized controlled trials to validate the broader use of FMT via PEC in treating UC^[19]^.

### Pretreatment strategies

Pretreatment protocols for FMT are crucial to optimize the engraftment of transplanted microbiota by clearing the residual contents of the gut and creating a more favorable environment for the new microbiome. Most studies, such as Sarbagili *et al*, Costello *et al*, and Rossen *et al*, used bowel lavage (2–3 L of macrogol or polyethylene glycol) to ensure a clean colon before the FMT procedure, enhancing microbial colonization and reducing interference from existing bacteria^[11,12,14]^.

However, some studies, such as Haifer *et al*, which used oral lyophilized FMT, did not require any specific pretreatment, suggesting that bowel preparation may not be as critical for non-invasive routes^[13]^. This raises questions about whether bowel preparation is necessary for all FMT methods or if certain delivery routes may bypass the need for intensive preparation. Similarly, Paramsothy *et al* did not use pre-FMT therapy, challenging the assumption that bowel preparation is always essential for successful outcomes^[37]^.

More unique approaches were reported by Cui *et al*, in which metoclopramide and esomeprazole were used before duodenal infusions, reflecting the role of specific preparations for different delivery routes^[38]^. Other studies, like Kunde *et al* and Angelburger *et al*, combined bowel lavage with antibiotics, suggesting that in some cases, reducing bacterial load through multiple mechanisms may enhance FMT efficacy^[36,39]^.

The variability in pretreatment protocols across studies reveals that, although bowel preparation is commonly used and generally beneficial, its necessity may depend on the route of FMT delivery and patient-specific factors. This variability suggests an opportunity to refine and individualize pretreatment approaches, potentially improving FMT outcomes by tailoring the protocol to the method of delivery and patient’s condition. It also highlights the need for further research to clarify when bowel preparation is essential and when it can be safely omitted without compromising efficacy.

### Microbiota modulation and clinical correlation

Several research studies have implored the impact of FMT on the gut microbiota composition in patients with UC, aiming to restore the microbial composition as a part of the treatment modality

In studies by Feng *et al* and Costello *et al* patients with UC exhibited dysbiotic gut microbiota before FMT, marked by reduced bacterial diversity and increased pro-inflammatory Proteobacteria and Bacteroidetes^[12,24]^. Post-FMT analysis revealed improved microbial diversity and a more stable microbiome resembling that of healthy individuals. The levels of beneficial bacteria, such as Firmicutes and Bifidobacterium, also increase, enhancing the gut barrier through their anti-inflammatory effects. These changes were correlated with clinical improvements in UC symptoms and endoscopic findings in some patients.

Studies by Narula *et al* and El Hage Chehade *et al* found that the pre-FMT microbial composition significantly affected the success of FMT in altering the microbiota and achieving remission^[26,31]^. Patients with higher microbial diversity before the procedure experienced longer-term remission than those with a severely disrupted microbiota. Shen *et al* emphasized the role of the intestinal microbiota in UC and the clinical benefits of probiotics and FMT, supporting the concept of microbiota reconstruction in disease management^[40]^.

A meta-analysis by Imdad *et al* highlighted that while FMT increased the overall microbiota diversity, concerns arose regarding the long-term persistence of these changes^[28]^. Some patients reverted to their pre-FMT dysbiotic state within months, correlating with UC relapse, suggesting that repeated FMT or additional therapies may be necessary to maintain a new microbial balance. Shi *et al* conducted a meta-analysis to confirm FMT’s positive effects of FMT on clinical outcomes and emphasized the need for standardized protocols to enhance efficacy^[41]^.

Thus, FMT has been shown to significantly improve the microbiota diversity of UC patients, bringing the microbial profile of UC patients closer to that of healthy subjects. However, the success and sustainability of these changes depend on the initial recipient microbiota and the stability of the transplanted microbiota.

### Patient preferences and perception

Patient preferences for FMT delivery methods are shaped by a combination of personal experience, disease characteristics, and cultural perceptions. Several studies have examined these preferences, revealing clear trends and shifts over time.

Zhong *et al* found that most FMT-naïve patients preferred enemas, while those with prior FMT experience showed a marked preference for colonic TET, with 42.3% choosing this method. Notably, 82.7% of patients – particularly those with UC – recommended TET^[42]^. Similarly, both adult UC patients and parents of children with UC have been shown to favor enemas or colonoscopy over nasogastric routes, indicating a general preference for lower GI delivery methods among this patient population^[43]^.

Several investigations have highlighted a growing trend toward colonoscopic FMT. One study^[44]^ reported that the most favored approach was a single sedated colonoscopy, followed by daily enemas for 5 days. Zeitz *et al* found that 49.7% of patients preferred colonoscopy, 21% chose enemas, and only 5.1% opted for nasogastric tubes^[45]^. By 2019, preferences had shifted further toward colonoscopy and capsule-based delivery, reflecting advancements in FMT technology and patient comfort^[46]^.

Yao Wei *et al* observed that, despite interest in odorless microbiota capsules, most patients still preferred colonoscopy due to discomfort associated with preparation and tube insertion for other methods^[47]^. In another study by Ya *et al*, 68% of participants believed TET better preserved their personal dignity compared to nasoduodenal tube delivery, which was preferred by only 32%^[48]^. These findings underscore the importance of both physical and psychological comfort in patient decision-making.

From the clinician’s perspective, gastroenterologists generally favor colonoscopic FMT, citing efficacy, infection risk, and aesthetic considerations^[49]^. However, each method has its limitations. Traditional enemas, for example, are restricted to the rectosigmoid colon and may not be suitable for UC patients who struggle to retain the solution. Frequent colonoscopic FMTs can be burdensome and costly due to the need for sedation and TET placement, while mid-gut delivery methods – such as endoscopic or nasogastric tube administration – are sometimes more manageable for certain patients^[50]^.

PEC represents an emerging modality, providing an efficient and accessible home treatment option. By aligning with natural gut function, PEC allows patients to adhere to FMT protocols at home and reduces the frequency of hospital visits^[19]^.

Across studies, patients have consistently shown a preference for lower GI routes (enema, colonoscopy, TET) over upper GI routes. Psychological discomfort with ingesting processed stool and cultural attitudes about feces and hygiene make less invasive and more familiar delivery methods more appealing. In summary, patient preferences for FMT delivery are dynamic and influenced by prior experience, perceived comfort, and practical considerations, with a clear trend toward lower GI and less invasive modalities.

While patient preference often aligns with less invasive and more familiar routes, current evidence suggests that clinical outcomes do not always directly correlate with these preferences. For example, studies have found that FMT delivered via oral capsules can achieve clinical remission rates comparable to colonoscopy, particularly in recurrent CDI, while offering greater convenience and patient acceptability^[51]^. Similarly, pooled analyses indicate that both upper and lower GI routes can be effective, with some trade-offs regarding convenience, invasiveness, and patient comfort. Thus, while preferred routes may enhance adherence and satisfaction, the choice of delivery method should balance patient preference with evidence-based efficacy, safety, and resource considerations.

### Social barriers and acceptance

Promoting FMT faces several barriers, including a limited understanding of its mechanisms, the “yuck factor,” and anesthesia concerns. The “yuck factor,” described by Schmidt^[52]^, includes feelings of disgust and fear, along with moral discomfort associated with fecal matter. Kahn *et al* noted that the term “fecal” can negatively affect perceptions, with many fearing ridicule if they share their FMT experience^[44]^.

Social stigma surrounding fecal material also raises concerns about cleanliness, infection risk, and worsening disease activity^[53,54]^. Similar concerns include discomfort with anonymous stool donors, anxiety over public perception, and reluctance to discuss FMT owing to its nature^[55]^. Better education on FMT could reduce anxiety and improve acceptance and outcome.

### Cost-effectiveness

The annual healthcare costs for UC in the United States range from $8.1 billion to $14.9 billion, reflecting a significant economic impact^[56]^. Direct costs per patient averaged over $3500, driven by hospitalizations during flare-ups and surgeries, with lifetime costs estimated around $405 496, covering inpatient, outpatient, pharmacy, and emergency services^[57,58]^.These considerable costs reflect the UC’s long-term financial burden on patients and the healthcare system. Additionally, travel costs and lost wages present further barriers to treatment for many patients^[59]^. FMT has shown favorable outcomes in UC treatment, with studies highlighting its cost-effectiveness. One study reported a substantial reduction in physician visits, hospital days, and medical expenses in the year following FMT, along with decreased societal costs and improved productivity due to reduced work absences^[60]^. For mild-to-moderate UC, combining conventional treatment with FMT improves health outcomes in quality-adjusted life years (QALYs), though cost-effectiveness varies based on remission rates and session frequency^[54]^. The majority (92.9%) of clinicians support establishing fee standards for FMT to improve its accessibility and affordability^[48]^. Among FMT delivery routes, oral capsules are the most cost-effective, requiring fewer resources and avoiding sedation costs^[61]^. While enemas are cheaper per treatment, repeated administrations increase long-term expenses^[37]^. Colonoscopy, though effective, is the costliest due to procedural and anesthesia fees^[62]^. Overall, oral capsules offer an optimal balance between efficacy and cost for UC management.

Another analysis of FMT costs revealed that the breakdown included costs associated with donor screening and drug preparation, which were $911.40 and $65.00, respectively. The overall cost of FMT varied according to the administration method: retention enema, $1021.41, flexible sigmoidoscopy at $1146.25; nasogastric tube insertion, $1329.30, and colonoscopy at $1592.75. In contrast, commonly used antibiotic treatments are more expensive, with fidaxomicin costing $3459.30 for a 10-day course, vancomycin taper at $1753.14, and vancomycin followed by rifaximin (2-week course) at $2285.14^[63]^. Although FMT may seem low-cost initially, the total expenses can be significant; however, they remain competitive because of their efficacy and lower overall costs compared with prolonged antibiotic treatments, indicating a need for further research on the cost-effectiveness of various FMT protocols.

In Taiwan, FMT was found to be more cost-effective than vancomycin and fidaxomicin in treating recurrent CDI in both general patients and those with IBD. The cost per QALY gained was approximately USD 39,356 for all patients and higher for patients with IBD^[64]^. Another study from Hong Kong based on publicly available data revealed the superior cost-effectiveness of FMT in IBD patients with rCDI relative to antibiotic treatment^[65]^. A recent study demonstrated that FMT is cost-effective for improving quality of life and reducing medical and societal costs in patients with moderate-to-severe IBD in a Chinese cohort^[60]^. To maximize health and economic benefits, FMT should be recommended based on specific baseline factors in different patient populations.

### Ethical concerns

Cultural attitudes towards fecal matter can significantly impact the acceptability of FMT. In some cultures, there may be strong stigmas associated with fecal material that hinder individuals from considering FMT as a viable treatment option. For instance, studies indicate that certain ethnic groups, such as Māori in New Zealand, show greater reluctance toward FMT than many other ethnic groups, stemming from historical and cultural factors that foster mistrust in medical practices^[66]^. This reluctance is further compounded by institutional bias, socioeconomic disparities, health literacy challenges, and a lack of cultural safety and representation in healthcare. Cultural concerns regarding FMT are particularly significant in China, where the historical use of human feces for medicinal purposes spans centuries. However, the modern application of FMT has encountered challenges due to past abuses in unregulated stem cell therapies that exploited vulnerable patients, prompting the government to enforce strict regulations requiring rigorous clinical trials for all medical interventions, including FMT^[67]^. Therefore, to effectively promote FMT in Middle Eastern countries, it is essential to consider specific social norms, traditions, customs, and religious beliefs to ensure its acceptance and proper implementation^[67]^.

Donor screening in FMT presents ethical challenges, particularly with regard to informed consent. The complex process involving extensive questionnaires and medical evaluations can overwhelm donors, hindering their understanding of the risks. Altruism and financial incentives may create a power imbalance, potentially pressuring vulnerable individuals to donate without full awareness. Similarly, patients’ stress and desperation can impair their ability to process risks, making them more susceptible to coercion or deception and raising concerns about autonomy and exploitation^[68,69]^. The donor screening process requires collecting sensitive health information, making privacy and confidentiality critical; however, there are inherent risks in managing this data. Any breach could lead to significant personal repercussions for donors, including discrimination or stigmatization based on their health status or lifestyle choices^[70]^. While anonymity is often maintained to protect donor identities, this can conflict with recipients’ desire for transparency regarding the donor’s health background. Balancing these interests is ethically challenging and requires careful consideration to respect both parties’ rights^[68]^. Furthermore, stringent donor eligibility criteria can limit individual autonomy by excluding potential donors based on factors unrelated to safety or effectiveness, such as dietary habits and lifestyle choices. This raises concerns about fairness and equity in access to donation opportunities^[68]^. Moreover, the practice of compensating stool donors raises ethical concerns regarding the commodification of human biological materials. While financial incentives can boost donations and alleviate shortages, they may also exploit economically disadvantaged individuals who might feel pressured to donate for monetary reasons rather than altruism^[71]^. These concerns closely parallel ethical debates in plasma and organ donation, where compensation has been shown to increase supply but also raises questions about exploitation, autonomy, and the commercialization of the human body. In plasma donation, for example, financial incentives have led to concerns about targeting vulnerable populations, while organ donation policies in most countries prohibit payment to avoid commodification and coercion^[72,73]^. Drawing on these precedents highlights the need for careful regulation and ethical oversight in FMT donor compensation practices

### Importance of donor-recipient matching

Variability in donor gut microbiota significantly influences the clinical outcomes of FMT. Research shows that only a portion of patients with UC experience positive responses to FMT, highlighting the importance of selecting suitable donors for successful treatment. Analysis of 656 fecal samples from both UC patients and healthy controls revealed notable differences in bacterial diversity and composition, with UC patients showing lower microbial richness and altered community structures. These findings indicate that greater microbial diversity in donors is linked to improved clinical outcomes for recipients, underscoring the need to refine the donor selection process to reduce variability in treatment responses among UC patients. This highlights the need for a personalized approach to FMT by ensuring that the chosen donors have microbiota capable of addressing the specific dysbiosis present in individual patients^[74]^. Furthermore, the concept of “super-donors” has emerged, referring to donors whose microbiota consistently yield better outcomes in recipients. Factors contributing to this phenomenon include the presence of keystone species and overall microbial richness. It has been suggested that both donor and recipient factors, including genetics and existing microbiota, significantly influence FMT success. Thus, advocating for a shift away from a “one stool fits all” approach towards more personalized FMT strategies that consider both donor and recipient microbiome profiles^[75]^.

### Dietary interventions

Diet plays a crucial role in the gut microbiome, affecting the risk and progression of IBD and is vital for patients undergoing FMT. Key recommendations include reducing gluten-based grains, refined sugars, and processed meats while increasing fresh fruits, vegetables, and fermented foods like yogurt. These dietary changes enhance intestinal barrier function, promote T-regulatory cells, and support healthy microbiota, thereby improving FMT outcomes^[12]^.

Eliminating processed and red meat while boosting the intake of fruits, vegetables, and fermented foods can enhance the effectiveness of FMT. However, cruciferous vegetables high in aryl hydrocarbon receptor (AhR) ligands should be avoided because of their potential negative effects on gut health. Focusing on nutrient-rich foods that provide beta-carotene, calcium, and magnesium is recommended, as poorly absorbed carbohydrates and fibers may worsen symptoms in patients with FMT.

### Dietary intervention after FMT in ulcerative colitis

FMT is a promising treatment for achieving remission in UC, particularly in mild-to-moderate cases. Diet significantly influences gut microbiota, which is essential for maintaining mucosal integrity and immune function. Dietary modifications after FMT can enhance the benefits of the transplanted microbiota and improve patient outcomes. However, FMT has not been widely adopted due to challenges such as insufficient long-term recovery data, legal restrictions, and stool bank availability^[12]^. Integrating dietary therapy post-FMT offers a novel approach to potentially enhance its effectiveness in managing UC.

#### Synergistic effects of dietary intervention

Another approach to dietary management after FMT is the 4-SURE diet, which focuses on reducing sulfur-containing proteins, sulfates, and sulfites while increasing fermentable fibers. This diet aims to minimize hydrogen sulfide (H_2_S) production, which can damage epithelial cells and worsen ulcerative colitis^[76]^. Research suggests that pairing FMT with an anti-inflammatory diet, such as the 4-SURE diet, leads to better clinical outcomes, including higher remission rates and improved long-term disease control^[12]^.

#### The impact of diet on gut microbiota

The gut microbiota is greatly influenced by diet, leading to changes in its composition and activity. A high-fiber diet stimulates the proliferation of advantageous bacteria, specifically Firmicutes and Bacteroidetes, which are frequently absent in individuals with UC^[4]^. Gut bacteria ferment fibers, especially soluble fibers, to generate short-chain fatty acids (SCFAs) such as butyrate, propionate, and acetate. These SCFAs possess anti-inflammatory characteristics and contribute to the preservation of mucosal well-being^[77]^.

Dietary changes are particularly important after FMT, as they optimize the gut environment for the transplanted microbiota. High-fiber diets are especially beneficial because they promote microbial diversity^[78,79]^ and the production of SCFAs such as butyrate and acetate, which have anti-inflammatory properties and aid in mucosal healing^[80]^. Soluble fibers found in foods like oats, barley, and certain fruits and vegetables are especially effective in enhancing these outcomes. Studies indicate that patients with UC with increased fiber intake after FMT experience fewer relapses and improved clinical outcomes^[12]^.

Prebiotics and probiotics are key dietary components that enhance the effectiveness of FMT. Prebiotics, such as those found in garlic, onions, and bananas, encourage the growth of beneficial gut bacteria, whereas probiotics, such as those found in fermented foods such as yogurt and kefir, help introduce additional helpful microorganisms^[81]^. Research has shown that combining prebiotics and probiotics with FMT improves symptom management and increases the likelihood of remission^[82]^.

**Figure 2.**
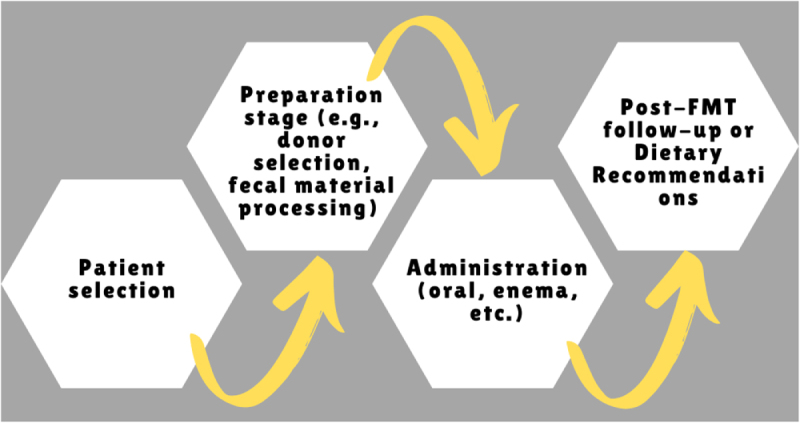
Flowchart of FMT process.

Figure 2 depicts a flowchart showing the step-by-step process for each FMT route, starting from patient selection to post-FMT follow-up or dietary recommendations.

#### Clinical evidence and implementation

Research has highlighted the significance of dietary adjustment following FMT. Huang *et al* found that patients on a high-fiber or low-FODMAP (Fermentable Oligosaccharides, Disaccharides, Monosaccharides, and Polyols) diet after FMT experienced better clinical outcomes compared to those on a standard diet, suggesting that dietary changes can enhance long-term remission and quality of life^[83]^. A low-FODMAP diet, which restricts certain fermentable carbohydrates, effectively alleviated symptoms in many patients with FMT^[84]^. However, it can be challenging and costly to maintain, potentially leading to nutrient deficiencies^[85]^. An alternative is the NICE-modified diet, which encourages regular meals, including psyllium husk fiber and spelt products, while limiting the consumption of fatty foods, onions, beans, and alcohol. Kedia *et al* suggested that combining multidonor FMT with an anti-inflammatory diet resulted in significantly higher rates of clinical response (65.7% vs. 35.5%), remission (60% vs. 32.3%), and deep remission up to 48 weeks (36.4% vs. 8.7%) compared to standard medical therapy, highlighting the potential role of dietary interventions alongside microbiome manipulation in IBD management^[86]^.

Given the complexity and individual variability in dietary needs, involving registered dietitians as part of the post-FMT care team is strongly recommended. Dietitians can personalize dietary plans based on patient-specific factors, monitor nutritional adequacy, and help manage potential food intolerances or deficiencies. Their expertise is crucial for optimizing dietary interventions, improving adherence, and ensuring that nutritional strategies effectively support both gut health and overall well-being in patients undergoing FMT.

### Future implications

Further research is needed to develop standardized protocols for fecal microbiota transplantation (FMT) in ulcerative colitis (UC), including pre-treatment and post-treatment dietary interventions, and consistent FMT administration methods. Clinical trials should focus on personalized microbiota matching, optimal treatment frequency, and long-term impacts on disease remission and mucosal healing.

Exploring cost-effective delivery methods, such as oral encapsulated formulations, could improve accessibility and patient adherence. Collaboration among dietitians, gastroenterologists, and microbiologists is essential to refine these protocols. Standardized evidence-based guidelines will ensure consistency across clinical settings, guide future clinical trials, and inform regulatory bodies and policymakers for better integration of FMT into routine patient care.

Table 3 provides a comparative overview of various FMT administration routes across several key parameters relevant to the treatment of UC.

**Table 3:** Comparative analysis of various FMT administration routes

Route of administration	Degree of invasiveness	Risks involved with administration	Self-administration possibility	Efficacy	Need of preparation before treatment	Cost	Patient preference
Transendoscopic enteral tubing	++	NA	No	High	Sedation	++	+++
Colonoscopy	+++	Perforation, bleeding	No	High	Bowel lavage/antibiotics/sedation	+++	+++
Oral capsules	-	Nausea, vomiting	Yes	Low	NA	+	+
Retention enema	+	NA	Yes	Low to moderate	Bowel lavage	+	++
Nasoduodenal route	++	Aspiration	No	Low	Sedation	+++	+
Nasojejunal route	++	Aspiration	No	Low	Sedation	+++	+
Percutaneous endoscopic cecostomy	++	Infection	Yes	High	NA	+++	+++

The notations (+, ++, +++) are intentional and denote relative magnitude or intensity (e.g., degree of invasiveness, risk, efficacy, cost, preference).

## Conclusion

In conclusion, this narrative review highlights the advantages of lower GI routes for FMT, such as colonoscopy and enema, which achieve higher clinical remission rates than upper gastrointestinal methods. These procedures are generally well tolerated with mild adverse events, and patients prefer them for their comfort. Oral capsules also offer viable and cost-effective treatment options for ulcerative colitis. Dietary interventions, such as low FODMAP diets and the use of prebiotics and probiotics, can enhance FMT outcomes by fostering a healthy microbiome and supporting long-term remission.

These findings advocate the development of standardized FMT protocols for UC management that combine effective delivery methods with dietary strategies to optimize patient care. While FMT shows promise as an adjunctive therapy, further research is needed to fully realize its potential and integrate it into routine practice. Future studies should examine key factors, such as donor selection, optimal dosage, preparation methods, antibiotic pretreatment, long-term safety, and the timing of FMT in relation to disease progression.

## Data Availability

Data are publicly available.
